# Radotinib Decreases Prion Propagation and Prolongs Survival Times in Models of Prion Disease

**DOI:** 10.3390/ijms241512241

**Published:** 2023-07-31

**Authors:** Yeong-Gon Choi, Byungki Jang, Jeong-Ho Park, Min-Woo Choi, Gong Yeal Lee, Dae Jin Cho, Hong Youp Kim, Hae Kyoung Lim, Won Jae Lee, Eun-Kyoung Choi, Yong-Sun Kim

**Affiliations:** 1 Ilsong Institute of Life Science, Hallym University, Youngdeungpo-gu, Seoul 07247, Republic of Korea; 2 Il Yang Pharm Co., Ltd., 37, Hagal-ro, 136beon-gil, Giheung-gu, Yongin-si 17096, Republic of Koreakimhongyub@ilyang.co.kr (H.Y.K.);; 3 Department of Biomedical Gerontology, Graduate School of Hallym University, Chuncheon 24252, Republic of Korea; 4 Department of Microbiology, College of Medicine, Hallym University, Chuncheon 24252, Republic of Korea

**Keywords:** radotinib, prion, drug repositioning, neurodegeneration

## Abstract

The conversion of cellular prion protein (PrP^C^) into pathogenic prion isoforms (PrP^Sc^) and the mutation of *PRNP* are definite causes of prion diseases. Unfortunately, without exception, prion diseases are untreatable and fatal neurodegenerative disorders; therefore, one area of research focuses on identifying medicines that can delay the progression of these diseases. According to the concept of drug repositioning, we investigated the efficacy of the c-Abl tyrosine kinase inhibitor radotinib, which is a drug that is approved for the treatment of chronic myeloid leukemia, in the treatment of disease progression in prion models, including prion-infected cell models, *Tga*20 and hamster cerebellar slice culture models, and 263K scrapie-infected hamster models. Radotinib inhibited PrP^Sc^ deposition in neuronal ZW13-2 cells that were infected with the 22L or 139A scrapie strains and in cerebellar slice cultures that were infected with the 22L or 263K scrapie strains. Interestingly, hamsters that were intraperitoneally injected with the 263K scrapie strain and intragastrically treated with radotinib (100 mg/kg) exhibited prolonged survival times (159 ± 28.6 days) compared to nontreated hamsters (135 ± 9.9 days) as well as reduced PrP^Sc^ deposition and ameliorated pathology. However, intraperitoneal injection of radotinib exerted a smaller effect on the survival rate of the hamsters. Additionally, we found that different concentrations of radotinib (60, 100, and 200 mg/kg) had similar effects on survival time, but this effect was not observed after treatment with a low dose (30 mg/kg) of radotinib. Interestingly, when radotinib was administered 4 or 8 weeks after prion inoculation, the treated hamsters survived longer than the vehicle-treated hamsters. Additionally, a pharmacokinetic assay revealed that radotinib effectively crossed the blood–brain barrier. Based on our findings, we suggest that radotinib is a new candidate anti-prion drug that could possibly be used to treat prion diseases and promote the remission of symptoms.

## 1. Introduction

Prion diseases are progressive, incurable, and fatal neurodegenerative disorders that are caused by the conversion of cellular prion protein (PrP^C^) into pathogenic prion isoforms (PrP^Sc^) and the mutation of *PRNP*. PrP^C^ is soluble and sensitive to proteases, but misfolded PrP^Sc^ is insoluble, forms aggregates, and is partially resistant to proteases, especially proteinase K (PK). Pathologically, prion diseases are characterized by spongiform changes, neuronal loss, glial cell activation, and misfolded PrP^Sc^ aggregation in the central nervous system [1]. These diseases are currently known to be transmissible zoonotic diseases, such as variant Creutzfeldt–Jakob disease (vCJD) (transmitted from bovines to humans) and chronic wasting disease (transmitted between cervids) [2]. Different human prion diseases have different characteristics based on their etiology: sporadic CJD (85–90% of CJD cases); acquired CJDs (less than 1% of CJD cases), including vCJD, iatrogenic CJD, and Kuru; and genetic CJDs (approximately 10–15% of prion disease cases) that occur due to prion gene (*PRNP*) mutation, including familial CJD, Gerstmann–Sträussler–Scheinker syndrome (GSS), and familial fatal insomnia (FFI). Disease progression depends on the type of CJD, and the incubation period can last from 1.5 to 40 years, and the typical duration of illness is 18 months after signs and symptoms appear [3,4,5]. Unfortunately, there are no available approaches for treating prion diseases or even delaying their progression. In addition, it is difficult to evaluate the efficacy of the drug because it is a rare disease (1.5 cases per 1 million/year). Therefore, the identification of medicines that may delay disease progression or treat these diseases is important.

Drug repositioning (or repurposing) is a strategy that involves the identification of new uses for (pre)approved, discontinued, or failed drugs in the treatment of untreatable diseases; this strategy has many advantages, including time and cost savings [6]. Accordingly, various drugs, such as imatinib mesylate (also known as STI571 or Gleevec, a tyrosine kinase inhibitor that is used to treat chronic myeloid leukemia), efavirenz (used for the chronic treatment of human immunodeficiency virus), quinacrine (used to treat malaria), and trazodone hydrochloride (anti-depressant drug), have been suggested as potential drugs for the treatment of prion diseases [7,8,9,10]. These drugs inhibit prion propagation or are neuroprotective, and they can result in prolonged survival times with or without improvements in pathological changes.

Radotinib hydrochloride (hereafter referred to as radotinib) is a c-Abl tyrosine kinase inhibitor that has been approved by the Korea Food and Drug Administration for the treatment of chronic myeloid leukemia [11,12]. Radotinib also effectively crosses the blood–brain barrier (BBB) and exerts neuroprotective effects in a Parkinson’s disease mouse model [13]. In addition, another c-Abl tyrosine kinase inhibitor, namely, imatinib mesylate, inhibits prion propagation and prolongs the survival times of scrapie-infected mice [7,14,15]. Therefore, it is worth investigating whether radotinib exerts anti-prion effects.

Here, we investigated the effects of radotinib in prion disease models using three approaches: (1) sustained PrP^Sc^-producing neuronal cell models, which provide a rapid means of identifying molecules capable of interfering with prion propagation; (2) the Prion Organotypic Slice Culture Assay (POSCA), which introduces a useful method to test anti-prion drugs. This approach allows for the examination of central nervous system pathologies in a complex cellular environment that closely resembles the intact brain. Furthermore, it enables a simplified incubation time bioassay, albeit with reduced precision, while requiring fewer animals [16,17]; and (3) 263K scrapie-infected golden hamster models, which is one of the well-defined in vivo prion models. In particular, hamsters received intragastric (IG) administration of radotinib in an early or late phase of infection, and then, survival times were monitored. We propose that radotinib could be repositioned for clinical trials in order to determine its efficacy for the treatment of prion diseases.

## 2. Results

### 2.1. Radotinib Decreases the Levels of Pathogenic Prion Isoforms in Prion-Infected Cells

**Figure 1:**
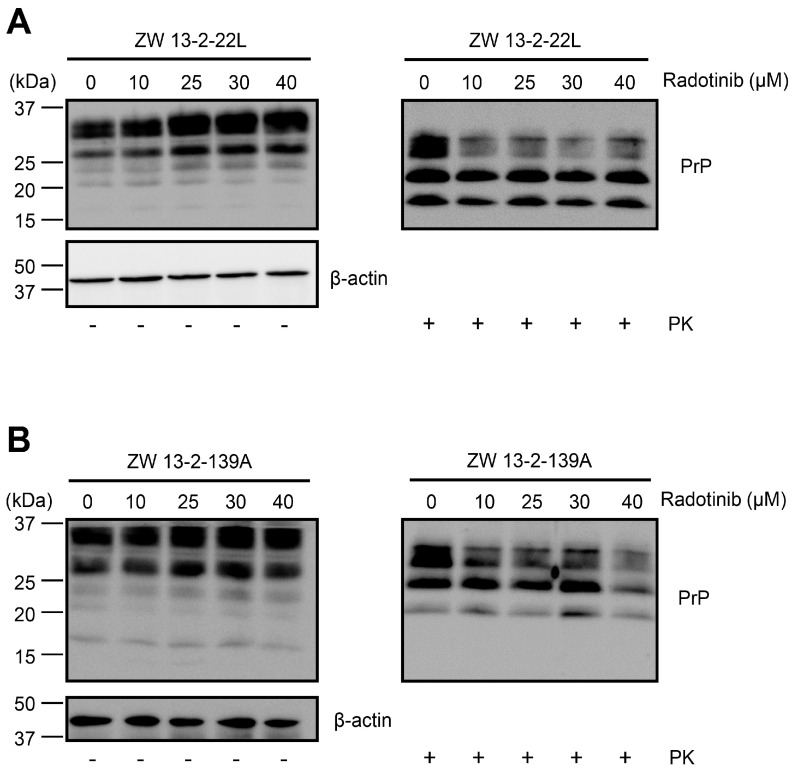
Radotinib treatment led to a mild reduction in PrP^Sc^ levels in neuronal cells with sustained prion production. ZW13-2-22L (**A**) and ZW13-2-139A (**B**) cells were incubated with radotinib (0–40 μM) for 24 h. Prion protein (PrP) levels after proteinase K treatment (PK, 5 μg/mL, 37 °C, 1 h incubation) or control were assessed by Western blotting with an anti-PrP antibody (3F10) [19]. Molecular masses in kDa are indicated on the left-hand side. β-actin was used as a loading control.

In our laboratory, we have generated a ZW13-2 neuronal cell line [18] that continuously produces PK-resistant prion proteins (PrP^Sc^) due to 22L and 139A scrapie agent infection. The cell model enables a relatively rapid assessment of the drug’s effect on PrP^Sc^ levels. To investigate the effect of radotinib on the uninfected and 22L or 139A scrapie-infected ZW13-2 neuronal cells (called ZW13-2-22L or ZW13-2-139A cells), the viability of these cells was monitored after exposure to radotinib (Supplementary Figure S1). No decrease in viability was observed after exposure to 40 μM radotinib; therefore, we used concentrations below 40 μM. We found that radotinib treatment led to a mild reduction in PrP^Sc^ levels in the ZW13-2-22L and ZW13-2-139A cells (Figure 1A,B, respectively), indicating that radotinib may inhibit PrP^Sc^ propagation or eliminate PrP^Sc^ in these cell models.

### 2.2. Radotinib Inhibits Prion Propagation in the Ex Vivo Cerebellar Tissue Slice Culture Model

**Figure 2:**
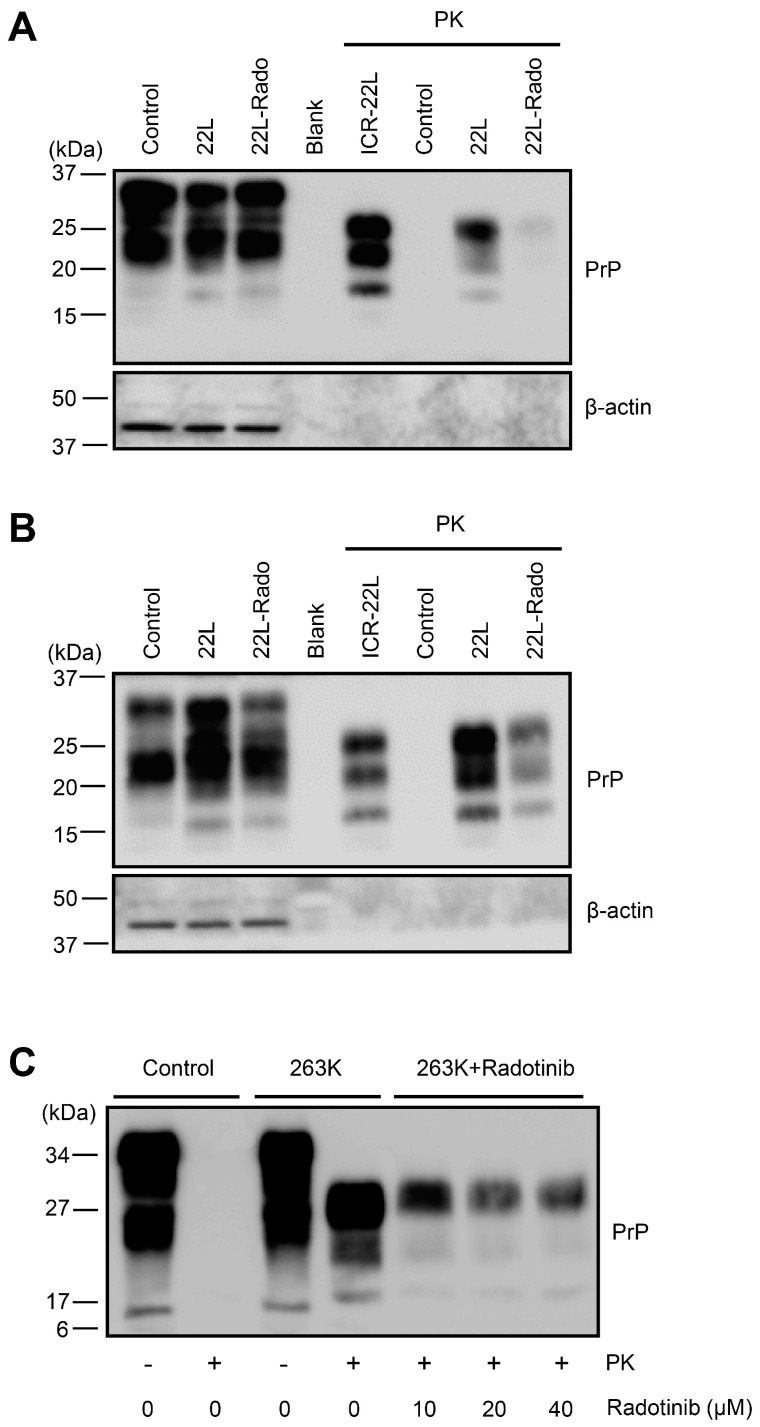
The effect of radotinib treatment on PrP^Sc^ deposition in 22L and 263K scrapie-infected cerebellar slice culture models. (**A**,**B**) Sliced *Tga*20 cerebellar tissues were exposed to 1% 22L scrapie agent for 1 h. Culture medium including radotinib (25 μM) was changed three times a week, and the cultures were maintained for 3 (**A**) or 5 (**B**) weeks. The cultured tissues were lysed using RIPA buffer, and then, PrP levels after proteinase K treatment (PK, 3 μg/mL, 37 °C, 1 h incubation) or control were assessed by Western blotting with an anti-PrP antibody (3F10). (**C**) Hamster cerebellar tissue slices were exposed to 1% uninfected or 263K scrapie-infected hamster brain homogenates for 1 h and then treated with radotinib (0–40 μM) for 5 weeks. PrP levels after PK treatment (1 μg/60 μg of total proteins, 37 °C, 1 h incubation) were assessed by Western blotting with an anti-PrP antibody (3F10). Molecular masses in kDa are indicated on the left-hand side. β-actin was used as a loading control.

Above, we showed the effect of radotinib in sustained PrP^Sc^-produced cell models. The limitation of the cell model seen above is its inability to fully represent prion diseases. Particularly, to observe the effects of the drug, it is necessary to monitor for a longer period of time. Therefore, to evaluate the effect of radotinib on PrP^Sc^ propagation, ex vivo *Tga*20 mouse (transgenic mice characterized by the 5–10-fold overexpression of mouse PrP) [20] and hamster cerebellar tissue culture models were established [17]. To determine whether cerebellar tissue culture with scrapie infection was successfully established, we confirmed that cerebellar cell death, accompanied by neuronal cell death and PrP^Sc^ accumulation, was increased in 22L scrapie-infected *Tga*20 cerebellar tissues compared to controls (Supplementary Figure S2). In these ex vivo models, we found that treatment with 25 μM radotinib reduced PrP^Sc^ propagation in 22L scrapie-infected *Tga*20 cerebellar tissue slices that were cultured for 3 or 5 weeks (Figure 2A,B). In addition, radotinib reduced PrP^Sc^ propagation in 263K scrapie-infected hamster cerebellar tissue slices in a dose-dependent manner (Figure 2C).

**Figure 3:**
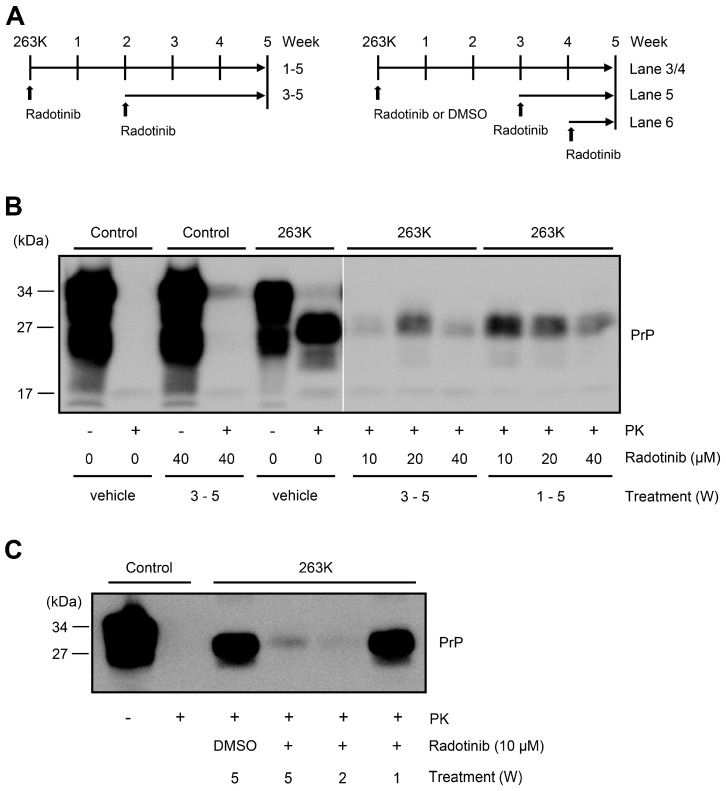
Late treatment with radotinib reduced PrP^Sc^ deposition in the 263K scrapie-infected hamster cerebellar slice culture model. (**A**) Schematic diagram of the experimental design. The left panel and right panel correspond to (**B**,**C**), respectively. (**B**) Uninfected and 263K scrapie-infected cerebellar tissue slices received radotinib treatment (0–40 μM) for 5 weeks (lanes 10–12) or the final 3 weeks of culture (lanes 3, 4, and 7–9). (**C**) 263K scrapie-infected cerebellar slice tissues were incubated with 10 μM radotinib for 5 weeks, the final 2 weeks of culture, or one week of culture. DMSO, Mock treatment. The cultured tissues were lysed, and then, the PrP levels after PK treatment (1 μg/50 μg of total proteins, 37 °C, 1 h incubation) were assessed by Western blotting with an anti-PrP antibody (3F10). W, week. Molecular masses in kDa are indicated on the left-hand side.

To determine whether radotinib exerts a practical effect during disease progression, we designed two experiments (Figure 3A). Hamster cerebellar tissue slices that were infected with the 263K scrapie strain (1) were cultured for 5 weeks in the presence of radotinib (Figure 3B, lanes 10–12) or (2) were cultured for 2 weeks in the absence of radotinib treatment followed by 3 weeks in culture medium containing radotinib, which was changed three times a week (Figure 3B, lanes 3, 4, 7–9). Then, the cerebellar tissue slices were lysed and subjected to immunoblotting, and we found that the levels of PrP^Sc^ were decreased after 5 weeks of treatment with radotinib in a dose-dependent manner (Figure 3B). Interestingly, later treatment with 10–40 μM radotinib also dramatically reduced the levels of PrP^Sc^. These observations indicate that radotinib can markedly inhibit, reduce, or delay the deposition of PrP^Sc^.

To expand on the study about whether treatment with radotinib 1 or 2 weeks after infection inhibits PrP^Sc^ propagation, 263K scrapie-infected hamster cerebellar tissue slices were cultured for 3 or 4 weeks, and then, radotinib was administered for the final weeks leading up to the 5-week endpoint. As shown in Figure 3C, treatment with radotinib in the final 2 weeks also effectively inhibited PrP^Sc^ propagation, and the effects were similar to 5 weeks of treatment; however, one week of treatment with radotinib did not reduce PrP^Sc^ propagation, similar to vehicle treatment of 263K-infected slices. Taken together, these results suggest that radotinib may exert anti-prion effects in vitro and ex vivo.

### 2.3. Radotinib Prolongs the Survival Times and Delays the Deposition of PrP^Sc^ in 263K Scrapie-Infected Hamsters

**Figure 4:**
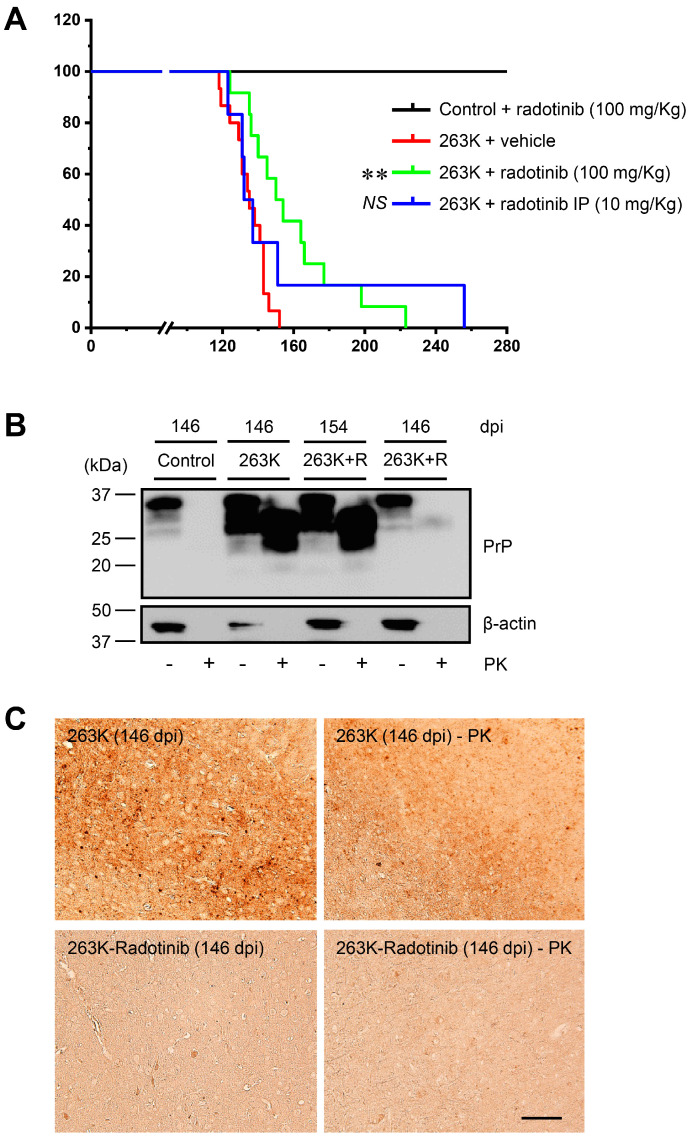
Intragastric administration of radotinib extends the survival time of 263K scrapie-infected hamsters and decreases PrP^Sc^ deposition. Uninfected hamsters (n = 4) received radotinib (100 mg/kg) as a control. Hamsters infected with the 263K scrapie strain received vehicle (n = 15) or radotinib (100 mg/kg/day for 6 days a week after 263K IP injection, n = 12) or intraperitoneally (10 mg/kg/day for 6 days a week 2 days postinoculation, n = 6). (**A**) The survival curve is presented as a Kaplan–Meier plot, and the log rank test was used for statistical analysis. ** *p* = 0.00217. NS, not significant. Vehicle, 0.5% CMC. (**B**) Brain homogenates from control hamsters that were sacrificed at 146 dpi (lanes 1 and 2), 263K scrapie-infected hamsters that were treated with vehicle and died naturally at 146 dpi (lanes 3 and 4), 263K scrapie-infected hamsters that were treated with radotinib and died naturally at 154 dpi (lanes 5 and 6), and 263K scrapie-infected hamsters that were treated with radotinib and sacrificed at 146 dpi (lanes 6 and 8) were incubated with or without PK (5 μg/mL) and then subjected to Western blotting. PrP levels were measured by Western blotting using an anti-PrP antibody (3F4). β-actin was used as a loading control. Molecular masses in kDa are indicated on the left-hand side. (**C**) Hamsters that were infected with the 263K scrapie strain, treated with vehicle, died naturally at 146 dpi (upper panels) as well as clinically healthy 263K scrapie-infected hamsters that were treated with radotinib and sacrificed at 146 dpi (100 mg/kg; bottom panels) were used for immunohistochemical staining. The anti-PrP antibody (3F4) was used. Scale bar = 200 μm.

Based on the in vitro and ex vivo results described above, we examined whether radotinib can prolong the survival time of hamsters that were intraperitoneally infected with the 263K scrapie strain. We administered radotinib (100 mg/kg in 0.5% carboxymethyl cellulose (CMC) sodium salt solution as a vehicle) once a day via the intragastric (IG) route for 6 days a week. As shown in Figure 4A, the survival time of the 263K scrapie-infected group was 135.1 ± 9.9 days postinoculation (dpi; range 118–152 dpi); however, the radotinib-treated group had a significantly prolonged survival time of 159.3 ± 28.6 dpi (range 124–223 dpi, *p* = 0.00217). Additionally, we injected radotinib via the intraperitoneal (IP) route 2 weeks after scrapie infection, which may exclude any possibility of interaction between the scrapie agent and drug, but there was no significant difference because only one hamster among six hamsters survived for 256 days (155 ± 50 dpi). This result suggests that the radotinib IG administration route is more advantageous for the treatment of prion diseases.

To determine whether IG administration of radotinib inhibits the propagation of PrP^Sc^ in the hamster model, we evaluated the levels of PrP^Sc^ in the brains of 263K scrapie-infected hamsters that were treated with radotinib and sacrificed at 146 dpi as a control (Figure 4, lanes 1, 2), 263K scrapie-infected hamsters that were treated with vehicle and died naturally at 146 dpi (Figure 4, lanes 3, 4), 263K scrapie-infected hamsters that were treated with radotinib and died naturally at 154 dpi (Figure 4, lanes 5, 6) and healthy 263K scrapie-infected hamsters that were treated with radotinib and sacrificed at 146 dpi (Figure 4, lanes 7, 8). PK-resistant PrP^Sc^ was deposited in high levels in the brains of 263K scrapie-infected hamsters that were treated with the vehicle (0.5% CMC) (Figure 4B, lane 4). Similarly, the PrP^Sc^ levels in the brains of the 263K scrapie-infected hamsters that were treated with radotinib and died at 156 dpi were also high (Figure 4B, lane 6). However, the PrP^Sc^ levels in the brains of the 263K scrapie-infected hamsters that were treated with radotinib and sacrificed at 146 dpi were markedly decreased (Figure 4B, lane 8) along with the total PrP levels (Figure 4B, lane 7).

Although the PrP^Sc^ levels were decreased, the total expression of PrP in the 263K scrapie-infected hamsters that were treated with radotinib (Figure 4B, lane 7) was similar compared to the control. Radotinib may inhibit or delay the deposition of PrP^Sc^ even though there were differences among 263K scrapie-infected hamsters that were treated with radotinib. Next, we performed immunohistochemical staining for PrP^Sc^ using 263K scrapie-infected hamsters that were treated with radotinib and sacrificed on 146 dpi. Compared to the brains of control-treated hamsters that were infected with the 263K scrapie strain, the total PrP and PK-resistant PrP^Sc^ levels were definitely lower in the brains of infected hamsters that were treated with radotinib (Figure 4C).

### 2.4. Wide Concentration Range of Radotinib Increases the Survival Time of 263K Scrapie-Infected Hamsters

**Figure 5:**
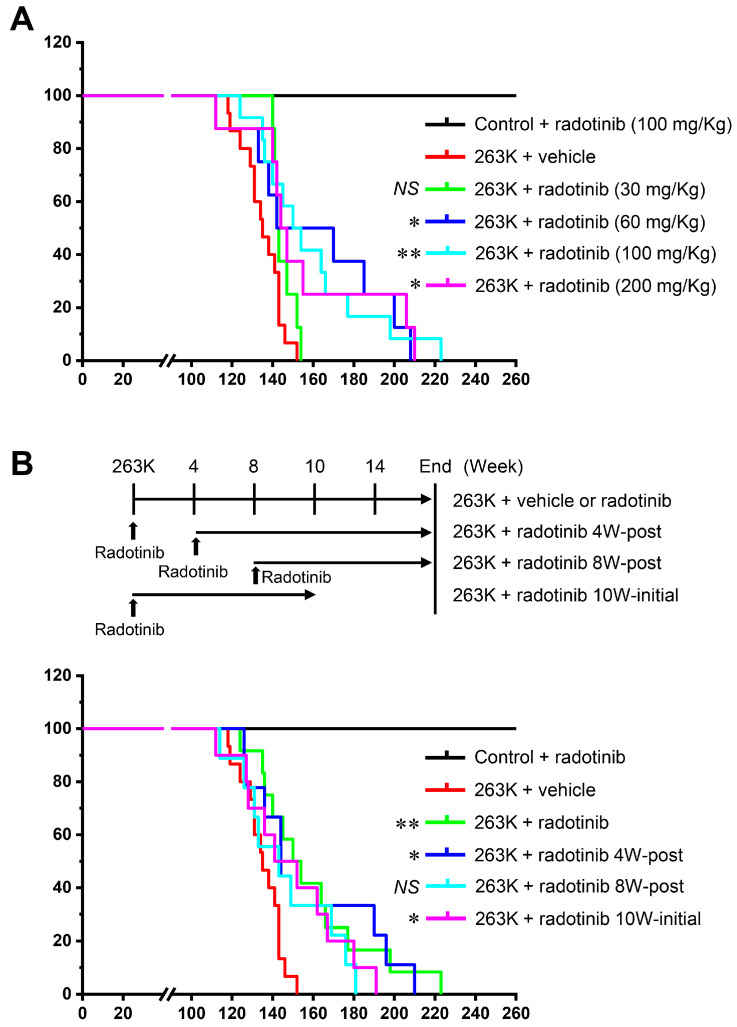
The effects of different doses and times of radotinib treatment on 263K scrapie-infected hamsters. (**A**) Hamsters were intraperitoneally infected with the 263K scrapie strain and received an IG injection of radotinib (30, 60, or 200 mg/kg). Kaplan–Meier plot shows significantly increased survival after 60 mg/kg radotinib treatment (n = 8), 200 mg/kg radotinib treatment (n = 8), and 100 mg/kg radotinib treatment (n = 12) but not after 30 mg/kg radotinib treatment (n = 8). (**B**) Schematic diagram of the experimental design (upper panel). Hamsters infected with the 263K scrapie strain were treated with vehicle (n = 15) or radotinib (100 mg/kg, n = 12), 4 weeks postinoculation (radotinib 4W-post, n = 9), 8 weeks postinoculation (radotinib 8W-post, n = 9), or for the initial 10 weeks (radotinib 10W-initial, n = 10). The survival curve is presented as a Kaplan–Meier plot, and the log rank test was used for statistical analysis (bottom panel). * *p* < 0.05, ** *p* < 0.01, NS, not significant.

Above, we showed the effect of radotinib when administered at different doses and for different time intervals in inhibiting PrP^Sc^ propagation in in vitro and ex vivo models. Therefore, we examined how various concentrations and times of radotinib treatment affected survival time in vivo. One hour after the IP infection with the 263K scrapie strain, hamsters were treated IG with 30, 60, or 200 mg/kg radotinib, and then, survival curves were generated; the results of each group were compared with those of the 100 mg/kg group (Figure 5A). The mean survival time of the 30 mg/kg radotinib group was 145.1 ± 5.3 dpi (range 140–154 dpi, *p* = 0.05848), that of the 60 mg/kg radotinib group was 161.0 ± 34.8 dpi (range 112–208 dpi, *p* = 0.03022), and that of the 200 mg/kg radotinib group was 157.0 ± 33.9 dpi (range 112–210 dpi, *p* = 0.01944). This result suggests that certain concentrations of radotinib are efficacious for prolonging the survival time of animals with prion diseases.

### 2.5. Late Treatment with Radotinib Delays Disease Endpoints of 263K Scrapie-Infected Hamsters

Next, we examined whether 4 or 8 weeks of late treatment with radotinib delays disease endpoints of 263K scrapie-infected hamsters. The mean survival times after 4 and 8 weeks of late treatment with radotinib were 158.0 ± 31.9 dpi (range 126–210 dpi, *p* = 0.02333) and 146.9 ± 23.7 dpi (range 114–181 dpi, *p* = 0.08559), respectively (Figure 5B). Additionally, we found that the initial 10 weeks of treatment with radotinib after 263K scrapie infection also significantly prolonged the survival time (149.6 ± 25.3 dpi, range 112–191 dpi, *p* = 0.0446). These results indicate that early treatment with radotinib is effective for the treatment of prion diseases.

### 2.6. Radotinib Crosses the Blood–Brain Barrier (BBB) of Hamsters

Previously, radotinib was shown to cross the BBB in a Parkinson’s disease mouse model [13]. However, it is unknown whether radotinib penetrates the BBB in hamster models. Firstly, we the efficacy of two c-Abl tyrosine kinase inhibitors, imatinib and nilotinib hydrochloride monohydrate (hereafter referred to as nilotinib), in inhibiting (or retarding) the deposition levels of PrP^Sc^ in hamster cerebellar tissue slice culture models. We confirmed that nilotinib reduced PrP^Sc^ levels, but imatinib had no effect (Supplementary Figure S3). Next, we investigated whether radotinib crosses the BBB of hamsters and compared its ability to do so with nilotinib. Nilotinib is a second-generation Abl inhibitor that was approved for the treatment of chronic myelogenous leukemia by the U.S. Food and Drug Administration (FDA) in 2007 [21]. Because the structures of radotinib and nilotinib are similar [22], nilotinib is a suitable agent for comparison in evaluating the BBB permeability of radotinib. We confirmed that radotinib (AUC_last_) crossed the BBB, and it was present in brain tissues at approximately 1.4 times higher levels than nilotinib after administration at 60 and 100 mg/kg (Table 1). The time to peak drug concentration (T_max_) of radotinib was slower than that of nilotinib, but the maximum concentration (C_max_) was similar. Notably, B/P ratio (%), AUC_last_ in brains treated with 60 mg/Kg and 100 mg/Kg radotinib was 13.7 ± 2.1 and 23.2 ± 15.5, respectively, which is a high absorption rate and is 2–3 times higher than nilotinib treatment. This result suggests that radotinib also effectively crosses the BBB in hamsters.

## 3. Discussion

Our study provides evidence of the potential anti-prion efficacy of radotinib in scrapie-infected neuronal cell models, brain tissue slice culture models, and, most interestingly, scrapie-infected hamster models. In these models, radotinib reduced PrP^Sc^ propagation, which may have resulted in prolonged survival times of prion-affected hamster models. To date, it has been a challenge to find or develop effective anti-prion drugs as well as drugs for the treatment of other neurodegenerative diseases, such as Alzheimer’s disease and Parkinson’s disease [23,24,25,26]. In particular, the duration of illness is typically shortened by approximately 18 months after signs and symptoms appear. Among the types of prion diseases, familial CJD accounts for 10–15% of cases [3,4]. In addition to complete disease treatment, prolonged clinical remission and delayed symptom onset need to be addressed. On the other hand, many compounds that are effective in preclinical tests are unsuitable for use in clinical trials due to low efficacy or unexpected toxicity. In addition, the development of new drugs for targeted disease treatment requires considerable time and costs [27]. Therefore, drug repositioning or repurposing methods are a very helpful strategy for investigating effective anti-prion drugs. In this regard, radotinib could be a drug candidate that could be quickly used in the treatment of prion diseases.

One of the major challenges to drug effectiveness is the blood–brain barrier (BBB) since it does not allow many drugs (either chemical compounds or proteins) to cross into the brain from the peripheral circulation. Abnormalities in PrP in the central nervous system are definite causes of prion diseases because PrP-deficient animal models do not progress to prion diseases [28]. Notably, radotinib effectively enters the mouse brain [13]. Here, we also confirmed the effective BBB penetration of radotinib and the absorption rate into the brain is better than nilotinib in hamsters (Table 1); therefore, radotinib may be active in directly inhibiting the conversion of PrP^C^ to PrP^Sc^ or the clearance of PrP^Sc^ in the brains of animal models (Figure 4) as well as in vitro and ex vivo models (Figure 1, Figure 2 and Figure 3). However, reductions in PrP^Sc^ levels are not always necessary for prolonged survival time. For example, the eIF2a-P inhibitors trazodone hydrochloride and dibenzoylmethane prevent neurodegeneration and significantly prolong survival without reducing the PrP^Sc^ levels in RML-infected hemizygous Tg37^+/−^ mice [9].

We showed that radotinib is effective in delaying neurodegeneration and prolonging survival time, although its effects vary (Figure 4 and Figure 5). Several radotinib-treated samples exhibited clinical health, the markedly low spread of pathological PrP^Sc^ in brain tissues, and delayed endpoints of disease, even in the context of 263K scrapie infection. Most importantly, late treatment with radotinib was also effective in ameliorating disease (Figure 5B). The survival times observed after 4 weeks of late treatment (158.0 ± 31.9 dpi after prion infection) were approximately 10 days longer than those observed after 8 weeks of late treatment with radotinib (146.9 ± 23.7 dpi). Additionally, an initial 10 weeks of treatment with radotinib resulted in prolonged survival times (149.6 ± 25.3 dpi), but the mean survival time was similar to that observed after 8 weeks of late treatment. Similarly, we also found that 2 weeks of late treatment with radotinib reduced (or inhibited) PrP^Sc^ deposition, but similar results were not observed after treatment with radotinib during only the final week of the cerebellar tissue slice culture models (Figure 3B). These results indicate that the early and continuous administration of radotinib is more effective in delaying disease progression. Possible or definite CJD patients are diagnosed at late time points after the onset of symptoms by biopsy, 14-3-3 protein and tau detection, and real-time quaking-induced conversion (RT-QUIC) [5]. In other words, in order to be a valuable therapeutic option, late drug treatment must have an effect on targeting the ongoing disease. Radotinib may meet this criterion.

Radotinib is a novel and second-generation c-Abl tyrosine kinase inhibitor whose chemical structure resembles that of imatinib mesylate and nilotinib, and it is approved for the chronic-phase treatment of chronic myeloid leukemia [12,22]. Interestingly, we found that radotinib and nilotinib reduced PrP^Sc^ accumulation, but imatinib had no effect in hamster cerebellar tissue slice culture models (Supplementary Figure S3). Previous studies have suggested that imatinib showed clearance of PrP^Sc^ in scrapie-infected Neuro2A cells and mice [7,14]. Since we only tested these compounds in hamster cerebellar tissue slice culture models, further studies are needed to investigate the anti-prion effects of various c-Abl tyrosine kinase inhibitors in different prion animal models.

Recently, c-Abl tyrosine kinase targeting has been considered a therapeutic approach for neurodegenerative diseases, including Alzheimer’s disease and Parkinson’s disease [29]. The synthetic neurotoxic prion fragment PrP106-126 activates c-Abl tyrosine kinase, and blocking or knocking down c-Abl protects neuronal cells from PrP106-126-induced mitochondrial dysfunction, reactive oxygen species production, and apoptosis [30]. On the other hand, reactive oxygen species (ROS) induce the localization of c-Abl tyrosine kinase to mitochondria, thereby mediating mitochondrial dysfunction, including the loss of mitochondrial transmembrane potential, depletion of ATP, and necrosis-like cell death [31]. Dynamin-related protein 1 (Drp1) is a critical factor in regulating mitochondrial dynamics. c-Abl tyrosine kinase phosphorylated Drp1, which enhances the GTPase activity of Drp1, and promotes Drp1-mediated mitochondrial fragmentation and cell death [32]. Recently, scrapie infection induced neuronal cell death accompanied by mitochondrial dysfunction, including the induction of mitochondrial ROS, the loss of mitochondrial membrane potential, and reduced ATP production [33]. In scrapie-infected mice, an imbalance in mitochondrial fusion and fission proteins such as Mfn1, Fis1, Mfn2, and Dlp1 was observed [34]. Additionally, the accumulation of endothelial nitric oxide synthase in mitochondria and downregulation of mitochondrial superoxide dismutase, cytochrome c, and ATP activity were observed in scrapie-infected mice [35]. Particularly, radotinib protects against α-synuclein preformed fibrils (PFF)-induced neuronal cell death and restores α-synuclein PFF-induced activation of Iba-1-positive microglia and GFAP-positive astrocytes in Parkinson’s disease models. Therefore, radotinib has the potential to act as a neuroinflammation suppressor [13]. Another tyrosine kinase inhibitor, STI571 (Gleevec or imatinib mesylate), causes the cellular clearance of PrP^Sc^ in prion-infected cells through STI571, which strongly activates the lysosomal degradation of PrP^Sc^ [14]. STI571 also protects neuronal cells from neurotoxic PrP fragment-induced apoptosis [15]. Based on these previous findings, we believe that radotinib may prevent dysregulated mitochondrial homeostasis, consequently leading to a delay in neuronal cell death caused by PrP^Sc^ toxicity while also ameliorating glial cell-mediated neuroinflammation. With a reduced accumulation of PrP^Sc^ (although the exact mechanism remains unknown), radotinib may prolong the survival time of prion-affected animal models.

In this study, we demonstrated for the first time that radotinib possesses anti-prion activity in in vitro, ex vivo, and in vivo prion models. Interestingly, late treatment with radotinib did not reduce its ability to decrease PrP^Sc^ deposition or prolong survival time. Therefore, we suggest that radotinib is a valuable drug candidate that could be rapidly used for the treatment of prion diseases. Moreover, our findings, along with the observations of other researchers, suggest strong associations between c-Abl tyrosine kinase (or c-Abl tyrosine kinase inhibitor) and pathological prion proteins. Further study of c-Abl tyrosine kinase inhibitors will advance the treatment of prion diseases.

## 4. Materials and Methods

### 4.1. Reagents

Unless stated otherwise, all chemicals and reagents were purchased from Sigma-Aldrich, Millipore, and Merck (Merck KGaA, Darmstadt, Germany), Duchefa Biochemie (Haarlem, The Netherlands), and Invitrogen, Gibco, Thermo Scientific, and Fisher Scientific (Thermo Fisher Scientific, Rockford, IL, USA).

### 4.2. Cytotoxicity of Radotinib

The mouse ZW13-2 hippocampal neuronal cell line was previously developed [18]. The cells were infected with scrapie strains (22L or 139A) and then subcultured; then, the sustained generation of PrP^Sc^ was confirmed. The cells were maintained in Opti-minimal essential medium (Invitrogen; Thermo Fisher Scientific, Rockford, IL, USA) supplemented with 10% fetal bovine serum (Biowest, Riverside, MO, USA) and 1% penicillin–streptomycin solution in a 5% CO_2_ incubator. Wild-type ZW13-2 cells (1 × 10^4^ cells), 22L scrapie-infected cells (called ZW13-2-22L cells, 1 × 10^4^ cells), and 139A scrapie-infected cells (called ZW13-2-139A cells, 1 × 10^4^ cells) were incubated with radotinib or dimethyl sulfoxide in 96-well plates for 24 h. Cell viability was assessed using the Cell Counting Kit (CCK)-8 (Dojindo, Kumamoto, Japan) with an ELISA plate reader.

### 4.3. Prion Organotypic Slice Culture Assay

Cerebellums were harvested from postnatal 12-day-old *Tga*20 mice [20] or postnatal 12-day-old golden hamsters, and a prion organotypic slice culture system was established as previously described [16,17]. The cerebellar tissues were sectioned to thicknesses of 300 μm using a Vibratome 3000 sectioning system (Technical Products International, St. Louis, MO, USA), and the sections were maintained in 6-well plates with membrane inserts (Millicell-CM inserts, 0.4 μm, 30 mm, Merck-Millipore, Billerica, MA, USA) in culture medium (100 mL 2 × minimal essential medium powder, 100 mL basal medium Eagle without glutamine, 100 mL horse serum, 4 mL GlutaMAX-I, 4 mL penicillin/streptomycin, 5.5 mL D-(+)-glucose solution and 86.5 mL ddH_2_O, pH 7.2–7.4) in a 5% CO_2_ incubator for 3 or 5 weeks. The medium was replaced 3 times per week. For prion inoculation, the cerebellar tissue slices were incubated with 1% 22L or 1% 263K scrapie agent in 6-well plates at 4 °C for 1 h and then placed on membrane inserts.

### 4.4. Western Blotting

Cells, tissue slices, and brain tissues were lysed using RIPA buffer. The proteins were separated with SDS–PAGE gels and transferred to nitrocellulose membranes. For the detection of target proteins, mouse monoclonal anti-PrP (3F4), mouse monoclonal anti-PrP (3F10) [19], and mouse monoclonal anti-β-actin (Sigma-Aldrich, Burlington, MA, USA) antibodies were used.

### 4.5. Immunohistochemistry

Neutral buffered formalin-fixed, paraffin-embedded brain tissue slices (5 μm thick) were used for immunohistochemical staining. The tissue slides were blocked with horse serum, incubated with an anti-PrP antibody (3F4), incubated with a biotinylated horse anti-mouse IgG secondary antibody using an ultrasensitive ABC peroxidase mouse IgG staining kit, and visualized with a 3,3′-diaminobenzidine (DAB) substrate kit (Thermo Fisher Scientific). After the sections were counterstained with hematoxylin, they were observed under a light microscope (BX51; Olympus, Southend-on-Sea, UK).

### 4.6. Animal Experiments

The animal experiments were approved by the Institutional Animal Care and Use Committees of Hallym University Medical Center (HMC 2019-0-0305-5-24). Golden hamsters (6 weeks of age, Japan SLC, Inc., Shizuoka, Japan) were intraperitoneally injected with 100 μL 1% brain homogenate from 263K scrapie-infected hamsters. Radotinib was dissolved in 0.5% CMC sodium salt solution, and intragastrically administered to the hamsters at doses of 30 to 200 mg/kg once daily for one week at the indicated times. After the terminal stage of scrapie agent incubation, when clinical manifestations of the disease were evident, the hamsters were sacrificed with CO_2_ gas, and brain tissues were harvested.

### 4.7. Pharmacokinetic Assay

Radotinib and nilotinib hydrochloride monohydrate were suspended in 0.5% CMC at a concentration of 60 mg/kg/10 mL or 100 mg/kg/10 mL, and these reagents were administered to 6-week-old male golden hamsters in a single dose by oral gavage. At 1, 2, 3, 4, 6, 8, and 24 h after administration, blood samples were collected by venipuncture of the abdominal vena cava under anesthesia with Zoletil 50 (Virbac)/Rompun (Bayer). All the blood samples were transferred to heparin tubes, and plasma was obtained by centrifugation at 3000 rpm for 10 min at 15 °C. After collecting the blood samples, the hamsters were transcardially perfused with PBS, and whole brains were isolated. The whole brains were weighed and homogenized in PBS (1:3 dilution). Then, 2-fold acetonitrile was added to the homogenized brain samples, and the mixtures were centrifuged at 14,000 rpm for 5 min at 4 °C. The supernatants were used to quantify the concentrations of radotinib and nilotinib. The concentrations of radotinib and nilotinib in the plasma and brain tissue samples were measured using LC–MS/MS. Pharmacokinetic parameters, including area under the curve from the time of dosing to the last measurable positive concentration (AUC_last_), maximum concentration (C_max_), time to reach C_max_ (T_max_), and terminal elimination half-life (T1/2), were calculated with the BA Calc 2007 analysis program (Ministry of Food and Drug Safety (MFDS), Republic of Korea).

### 4.8. Statistical Analysis

The probability of statistically significant differences between experimental groups is presented in each figure legend or table legend using OriginPro 2023 software (OriginLab Corporation, Northampton, MA, USA) or the program R (version 4.0.2).

## Figures and Tables

**Table 1:** Pharmacokinetic (PK) parameters in the plasma and brain tissues of hamsters after treatment with radotinib and nilotinib.

PK Parameters	Radotinib				Nilotinib			
	Plasma		Brain		Plasma		Brain	
	60 mg/kg	100 mg/kg	60 mg/kg	100 mg/kg	60 mg/kg	100 mg/kg	60 mg/kg	100 mg/kg
AUC_last_ (ng·h/mL)	1558.8 ± 176.0	1978.2 ± 466.8	215.5 ± 55.8	416.9 ± 183.7	1951.2 ± 675.9	3888.0 ± 771.9	147.4 ± 54.3	292.8 ± 49.2
C_max_ (ng/mL)	461.3 ± 115.0	554.1 ± 98.8	63.5 ± 20.9	81.7 ± 6.7	650.2 ± 219.1	1292.5 ± 174.4	51.0 ± 19.6	84.0 ± 11.3
T_max_ Median (range, h)	6.0 ± 0.0	6.0 ± 1.15 (6.0–8.0)	6.0 ± 0.0	6.0 ± 0.0	2.0 ± 0.6 (2.0–3.0)	3.0 ± 0.6 (2.0–3.0)	2.0 ± 1.0 (1.0–3.0)	3.0 ± 1.0 (2.0–4.0)
T_1/2_ (h)	NC	NC	NC	3.3 ^‡^	1.0 ± 0.4	1.2 ± 0.2	1.4 ± 0.3	1.4 ± 0.7
B/P ratio (%), AUC_last_			13.7 ± 2.1 ***	23.2 ± 15.5 ***			7.5 ± 0.3	7.6 ± 0.5
B/P ratio (%), C_max_			13.7 ± 2.3 **	14.9 ± 1.4 ^††/†††^			7.8 ± 0.6	6.5 ± 0.6

The values are shown as the mean ± SD from the plasma (P) and brain (B) of three golden hamsters. ^‡^ A single value was obtained from one hamster since the parameter was not assessed in two other hamsters. NC, not calculated. *** *p* < 0.001 compared to the B/P ratio (%) and AUC_last_ of the groups treated with both concentrations of nilotinib. ** *p* < 0.01 compared to the B/P ratio (%) and C_max_ of the groups treated with both concentrations of nilotinib. ^††^ *p* < 0.01 compared to the B/P ratio (%) and C_max_ of the 60 mg/kg nilotinib group. ^†††^ *p* < 0.001 compared to the B/P ratio (%) and C_max_ of the 100 mg/kg nilotinib group. By using the program R (version 4.0.2), both the B/P ratio (%) and AUC_last,_ as well as the B/P ratio (%) and C_max,_ were over 95% in the Shapiro test (normality test), but only the B/P ratio (%) and C_max_ were over 95% in the Bartlett test (homogeneity of variance). Thus, after the Box–Cox transformation of the B/P ratio (%) and AUC_last_ values, both the Shapiro test and the Bartlett test were performed again (*p* > 0.05 in both the normality and the homogeneity of variance tests). Using the B/P ratio (%), C_max_ values, and the transformed B/P ratio (%) and AUC_last_ values, statistical analysis was carried out by one-way ANOVA. The significance was determined using the post hoc test (Tukey HSD).

## Data Availability

The datasets used and/or analyzed during the current study are available from the corresponding author upon reasonable request.

## Supplementary Materials

The following supporting information can be downloaded at: https://www.mdpi.com/article/10.3390/ijms241512241/s1.
