# Supplementary material for: Radotinib Decreases Prion Propagation and Prolongs Survival Times in Models of Prion Disease
Source: Int J Mol Sci. 2023 Jul 31;24(15):12241. doi: 10.3390/ijms241512241 (PMC10419185; doi:10.3390/ijms241512241)
Supplement: Supplementary file 1 [file ijms-24-12241-s001.zip › ijms-2489475-supplementary.pdf]

Supplementary Figure S1

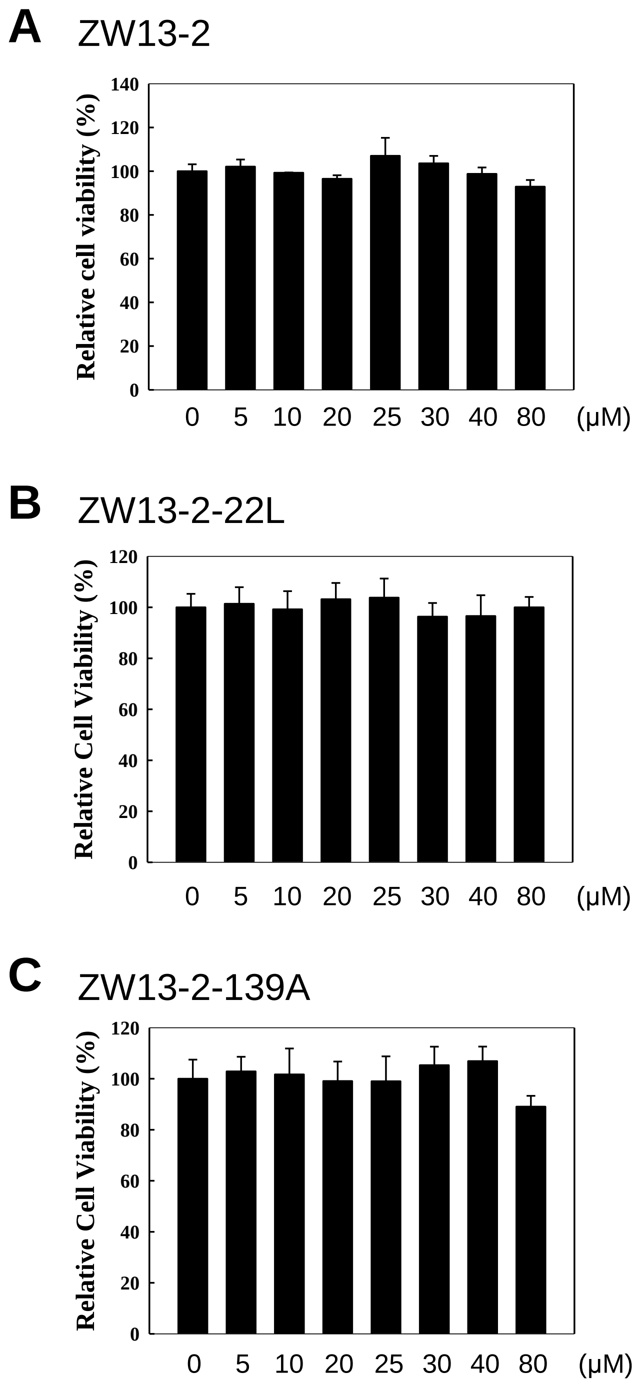

Supplementary Figure S1. Viability of ZW13-2 cells, ZW13-2-22L cells, and ZW13-2-139A cells. Wild-type ZW13-2 cells (**A**), ZW13-2-22L cells (**B**), and ZW13-2-139A cells (**C**) were treated with radotinib (0, 5, 10, 20, 25, 30, 40 or 80 μM) for 24 hours. Cell death was analyzed by CCK-8 assay. The cell viability is represented by bars (mean ± SEM, n = 3).

Supplementary Figure S2

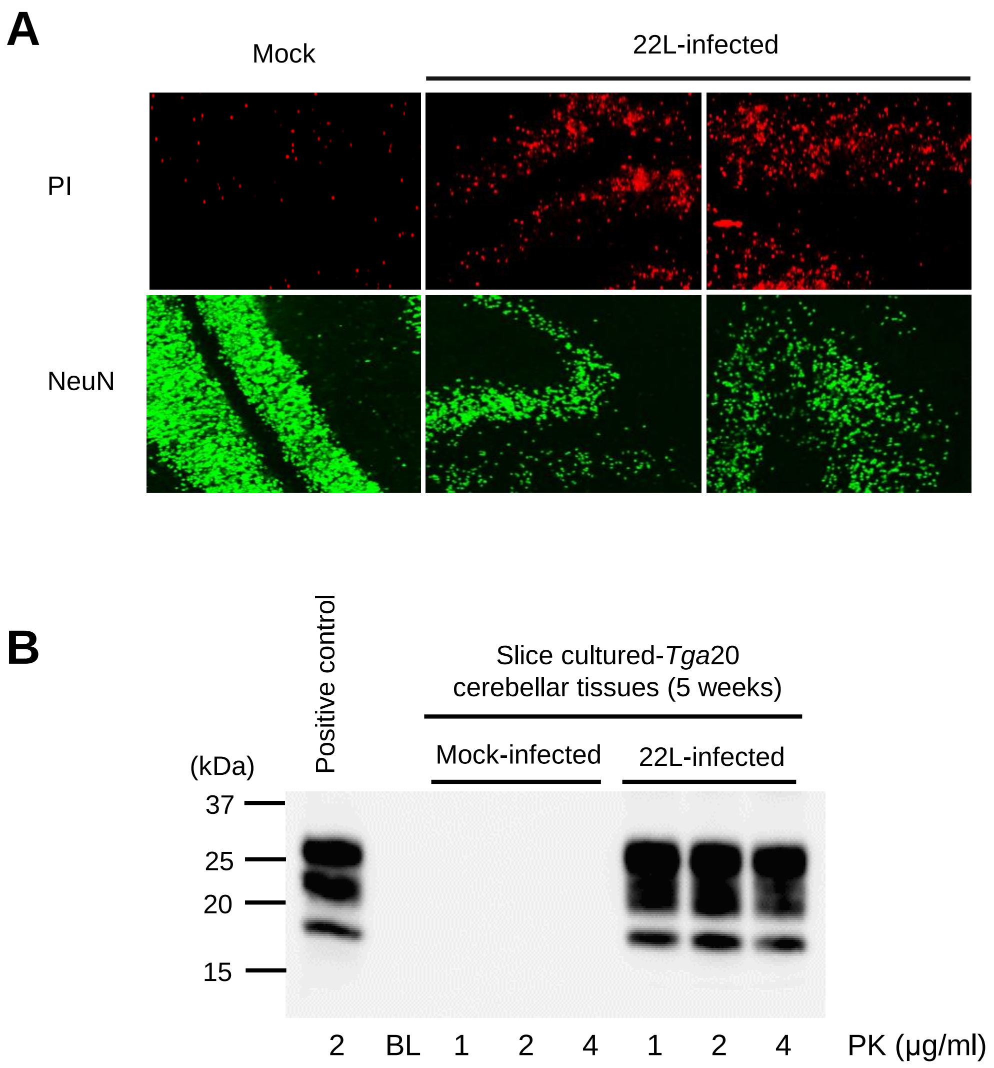

Supplementary Figure S2. PK-resistant pathogenic prion protein in the 22L scrapie-infected *Tga*20 cerebellar slice tissue culture model. (**A**) Sliced cerebellar tissues were exposed to 1% 22L scrapie-infected brain homogenates (BH), and then were cultured for 5 weeks. Cultured tissues were stained with propidium iodide (PI) or GFP-labeled anti-NeuN antibody. (**B**) Cultured cerebellar tissues were homogenized in RIPA buffer, and then lysates were digested with proteinase K (PK, 1-4 μg/ml) at 37°C for 1 hour. Prion protein was assessed by Western blotting with anti-PrP antibody (3F10). Positive control, BH from 22L-infected ICR mouse. BL, blank. Molecular masses in kDa are indicated on the left-hand side.

Supplementary Figure S3

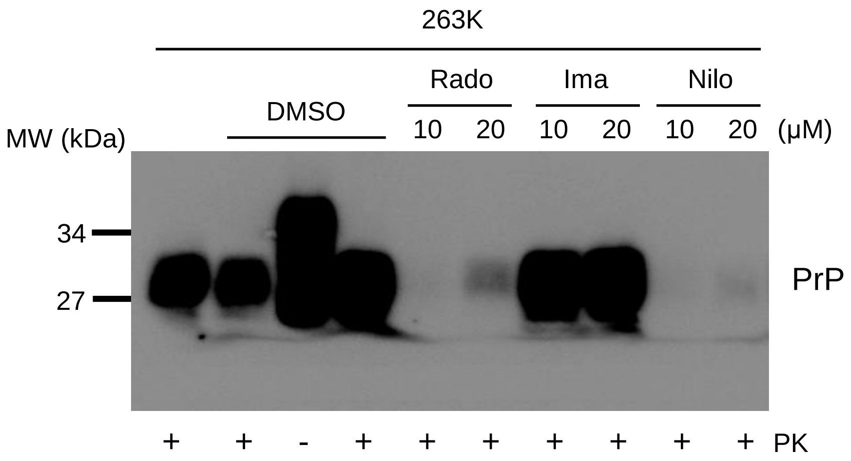

Supplementary Figure S3. Radotinib and nilotinib, but not imatinib, inhibit the deposition levels of PrP^{Sc} in hamster cerebellar slice tissues culture. Hamster cerebellar slice tissues inoculated with 1% 263K prion-infected brain homogenates were incubated with radotinib (Rado), imatinib (Ima), or nilotinib (Nilo) for 5 weeks. Proteinase K (1 μg/50 μg of proteins) was applied to each sample at 37°C for 1 hour. A total of 50 μg of protein was separated and immunoblotted with the anti-PrP antibody (3F10). Molecular masses in kDa are indicated on the left-hand side.
